# The affective modulation of motor awareness in anosognosia for hemiplegia: Behavioural and lesion evidence

**DOI:** 10.1016/j.cortex.2014.08.016

**Published:** 2014-12

**Authors:** Sahba Besharati, Stephanie J. Forkel, Michael Kopelman, Mark Solms, Paul M. Jenkinson, Aikaterini Fotopoulou

**Affiliations:** a King's College London, Institute of Psychiatry, UK; b Department of Psychology, University of Cape Town, South Africa; c Clinical, Educational & Health Psychology, Division of Psychology & Language Sciences, University College London, UK; d King's College London, Department of Neuroimaging, Natbrainlab, Institute of Psychiatry, UK; e Department of Psychology, School of Life and Medical Sciences, University of Hertfordshire, UK

**Keywords:** Anosognosia, Motor awareness, Emotion, Insula, Basal ganglia, VLSM

## Abstract

The possible role of emotion in anosognosia for hemiplegia (i.e., denial of motor deficits contralateral to a brain lesion), has long been debated between psychodynamic and neurocognitive theories. However, there are only a handful of case studies focussing on this topic, and the precise role of emotion in anosognosia for hemiplegia requires empirical investigation. In the present study, we aimed to investigate how negative and positive emotions influence motor awareness in anosognosia. Positive and negative emotions were induced under carefully-controlled experimental conditions in right-hemisphere stroke patients with anosognosia for hemiplegia (*n* = 11) and controls with clinically normal awareness (*n* = 10). Only the negative, emotion induction condition resulted in a significant improvement of motor awareness in anosognosic patients compared to controls; the positive emotion induction did not. Using lesion overlay and voxel-based lesion-symptom mapping approaches, we also investigated the brain lesions associated with the diagnosis of anosognosia, as well as with performance on the experimental task. Anatomical areas that are commonly damaged in AHP included the right-hemisphere motor and sensory cortices, the inferior frontal cortex, and the insula. Additionally, the insula, putamen and anterior periventricular white matter were associated with less awareness change following the negative emotion induction. This study suggests that motor unawareness and the observed lack of negative emotions about one's disabilities cannot be adequately explained by either purely motivational or neurocognitive accounts. Instead, we propose an integrative account in which insular and striatal lesions result in weak interoceptive and motivational signals. These deficits lead to faulty inferences about the self, involving a difficulty to personalise new sensorimotor information, and an abnormal adherence to premorbid beliefs about the body.

## 1 Introduction

Neurological disturbances of body awareness provide a useful way of investigating the bodily self; a fundamental facet of self-consciousness (Gallagher, 2000). Anosognosia for hemiplegia (AHP; i.e., the denial of motor deficits contralateral to a brain lesion) is a prototypical example of a disturbance in body awareness. AHP occurs more frequently following right perisylvian lesions, and less often following left-hemisphere perisylvian lesions (Cocchini, Beschin, Cameron, Fotopoulou, & Della Sala, 2009). AHP can take various clinical forms, ranging from blatant denial of limb paralysis and associated delusional beliefs to milder forms of motor unawareness (see Fotopoulou, 2014; Jenkinson, Preston & Ellis, 2011; Marcel, Tegnel & Nimmo-Smith, 2004). Although the exact aetiology of AHP remains debated, the clinical variability of AHP suggests that it is a multifaceted and heterogeneous phenomenon (Fotopoulou, 2014; Marcel, Tegner, & Nimmo-Smith, 2004; Orfei et al., 2007; Vocat, Staub, Stroppini, & Vuilleumier, 2010). Accordingly, explanations have varied from selective deficits in motor planning, to multi-factorial accounts involving both basic sensorimotor and higher-order cognitive deficits (see Fotopoulou, 2014; Jenkinson & Fotopoulou, 2010 for reviews). These cognitive deficits have been associated with either particular lesion sites such as the premotor cortex (Berti et al., 2005) and the insula (Karnath, Baier & Nagele, 2005), or involvement of a more varied pattern of cortical and subcortical regions and their connections (Fotopoulou, Pernigo, Maeda, Rudd, & Kopelman, 2010; Moro, Pernigo, Zapparoli, Cordioli & Aglioti, 2011; Vocat et al., 2010).

One facet of AHP that has received less empirical attention, despite a long history of clinical observations and theoretical debates (Bisiach & Geminiani, 1991; Weinstein & Kahn, 1955), is the role of emotional factors. On clinical examination, patients typically manifest some degree of blunted affect or ‘indifference’ for their paralysis and its consequences. This indifference (anosodiaphoria, Babinski, 1914) can exist with or without concomitant explicit denial of deficits. On the contrary, depressive symptoms and ‘catastrophic reactions’ (sudden influx of strong, negative feelings and related behaviours; Goldstein, 1939) are encountered rarely. Moreover, there are some clinical indications that as unawareness decreases over time, depressive symptoms begin to emerge in patients who were previously emotionally unresponsive towards their paralysis (Besharati, Kopelman, Avesani, Moro, & Fotopoulou, 2014; Fotopoulou, Rudd, Holmes & Kopelman, 2009; Kaplan-Solms & Solms, 2000). Exceptionally, some patients with or without explicit denial of deficits have been noted to show a strong hatred towards their paralysed limbs (misoplegia; Critchley, 1974), or a disproportionate exasperation with irrelevant, minor disappointments, despite their apparent indifference for their paralysis (Fotopoulou & Conway, 2004; Kaplan-Solms & Solms, 2000; Weinstein & Kahn, 1950).

Some authors have argued that this lack of affect, or misattribution of negative emotions, is caused by purely psychogenic ‘defence’ mechanisms. According to the now classic theory of Weinstein and colleagues (e.g. Weinstein, 1991; Weinstein & Kahn, 1955), denial and related premorbid coping mechanisms prevent patients from explicitly acknowledging their paralysis, and self-attributing the associated negative emotions. Alternatively, this lack of emotional reactivity has been considered to be the direct consequence of damage to the right (frontal) hemisphere, regarded by some authors as specialised for the processing of negative, withdrawal-related emotions (Davidson, 2001; see Gainotti, 2012 for review). However, neither of these two approaches has been fully supported by empirical evidence. Specifically, the psychodynamic account of AHP fails to explain the relative neuroanatomical and behavioural specificity of anosognosic behaviours (Bisiach & Geminiani, 1991; Heilman & Harciarek, 2010). The ‘valence’ hypothesis has similarly not been supported in the literature; although patients with AHP do typically score lower than control patients in self-report measures of depression and anxiety (e.g., Fotopoulou et al., 2010), more sensitive investigations have shown that they do not differ from controls groups in their ability to experience such emotions (Turnbull, Evans, & Owen, 2005; Vocat et al., 2010). They also show appropriate, negative emotional reactions to their deficits when the latter are evoked implicitly (Fotopoulou et al., 2010; Nadrone, Ward, Fotopoulou, & Turnbull, 2007). Thus, it appears that the relation between AHP and emotion is more complex than suggested by either the psychodynamic or the valence hypothesis.

More generally, such rigid distinctions between purely psychodynamic and neurocognitive explanations have been challenged recently (Fotopoulou, 2012) and integrative accounts of AHP have been put forward (Fotopoulou, 2010; Turnbull et al., 2005; Turnbull & Solms, 2007; Vuilleumier, 2004; see also Turnbull, Fotopoulou & Solms, 2014). According to such theories, complex imbalances between cognition and motivation may be caused directly by damage to insular, striatal, or limbic regions that have recently been found to be selectively associated with AHP (Fotopoulou et al., 2010; Moro, Pernigo, Zapparoli, Cordiolo, & Aglioti, 2011; Vocat et al., 2010). For example, Vuilleumier and colleagues have suggested that damage to the basal ganglia may obstruct the “discovery” of deficits, as patients have reduced affective drive to respond to errors and revise beliefs based on new perceptual evidence (Vocat, Saj, & Vuilleumier, 2013; Vuilleumier, 2000, 2004). Similarly, within a computational framework, Fotopoulou and colleagues have suggested that insular and basal ganglia damage may lead to weak and imprecise signals about the physiological condition of one's body. This leads to aberrant ‘top-down’ inferences about bodily states, and difficulties in affectively personalising new sensorimotor information (Fotopoulou, 2014).

Taken together, these accounts suggest that the lack or misattribution of negative emotions in AHP relates to impairments in higher-order cognition, rather than to primary deficits in emotional processing. This ‘top-down’ perspective is consistent with a relatively neglected facet of AHP, namely, the fluctuations of awareness based on the emotional or social context in which awareness is probed. For instance, Kaplan-Solms and Solms (2000), see also Ross & Rush (1981); Starkstein & Robinson (1988); Turnbull, Jones, & Reed-Screen (2002) have shown that when themes of loss are explored during psychotherapeutic sessions – particularly when such loss is apparently unrelated to their disabilities – transient awareness and depressive episodes can be experienced by patients that are otherwise stably anosognosic. Marcel et al. (2004) have further shown that awareness may increase in some patients when they are asked about their disabilities in an emotional, conspiratory manner, or from the perspective of the examiner (see also Fotopoulou, 2014; Fotopoulou, Rudd, Holmes, & Kopelman, 2009). Notwithstanding the theoretical interest of these observations, to our knowledge there is no systematic, experimental investigation of the moderating role of emotional and social context in AHP.

Accordingly, we aimed to investigate the relation between emotion and motor awareness in AHP. To this end, we recruited right-hemisphere stroke patients with AHP and control patients without AHP, and assessed motor awareness before and after providing positive and negative feedback about performance on a standardised cognitive test (the Hayling Test; Burgess & Shallice, 1997). The task includes components of varied difficulty that we could match with the valence of the provided feedback to generate realistic conditions of positive and negative feedback. Moreover, it is unrelated to motor abilities so we could test the role of emotion on motor awareness, uncomplicated by ‘bottom-up’ sensorimotor signals and the patients' explicit or implicit feelings about their motor abilities. Based on the idea that patients with AHP have lost the ability to use signals from their own body to make related inferences about their current bodily state (Fotopoulou, 2014; see also above), our main aim was to test whether the ‘top-down’ experimental induction (by verbal, social feedback) of negative feelings about oneself could improve awareness of one's motor disabilities. We expected patients with AHP to show increased awareness of their deficits following negative feedback compared with positive feedback, while such effects were not expected in the control group. Furthermore, in order to ensure that the experimental feedback had induced the desired emotions in patients, we measured patients' self-reported emotional state following each condition of the main task. If patients with AHP were capable of experiencing negative emotions, we expected negative feedback to lead to more negative feelings than positive feedback in both patient groups.

Lastly, we examined whether lesions to critical cortical (premotor and the insular cortex) and subcortical (basal ganglia and limbic structures) areas would be associated with increased unawareness scores, as in previous studies (Berti et al., 2005; Fotopoulou et al., 2010; Karnath et al., 2005; Moro et al., 2011). Contrary to such lesion subtraction investigations, however, we used a voxel-based lesion-symptom mapping (VLSM) approach (Bates et al., 2003; Rorden, Karnath, & Bonilha, 2007). This advanced method characterises the statistical relationship between tissue damage and behaviour on a voxel-by-voxel basis, regardless of the classification of patients into categorical groups, or implementing a cut-off for pathology (Bates et al., 2003). We also used this method to identify the brain regions associated with a change in motor awareness induced by our experimental task, which according to our hypothesis should include the insular cortex and basal ganglia structures (Fotopoulou, 2014; see also above). While the first clinico-anatomical correlation has been investigated before in the literature, to our knowledge, only two previous studies have investigated the association between behaviour on carefully-controlled experimental conditions and neuroanatomical data (Fotopoulou et al., 2010; Moro et al., 2011), and no study has examined this association in relation to emotion.

## 2 Methods

### 2.1 Patients

Twenty-five, adult neurological patients with right-hemisphere lesions were recruited from consecutive admissions to an acute, stroke-rehabilitation ward. Inclusion criteria were: (i) right-hemisphere lesion as confirmed by clinical neuroimaging; (ii) contralateral hemiplegia; and (iii) <4 months from symptom onset. Exclusion criteria were: (i) previous history of neurological or psychiatric illness; (ii) <7 years of education; (iii) medication with severe cognitive or mood side-effects; (iv) language impairments that precluded completion of the study assessments. Of the initial 25 patients screened, nine could not be tested due to time constraints (*n* = 4), fatigue or poor concentration (*n* = 3), and early discharge (*n* = 2). Thus, a total of 16 patients took part in the study (nine women; mean age = 68.19, SD = 14.27 years, age range: 41–88). Two additional sets of patients were recruited subsequently in order to test (see section 2.4): (i) a control condition in which the order of experimental conditions was reversed (*n* = 2; two women with AHP, 82 and 90 years of age); and (ii) the specificity of the effect to motor awareness (*n* = 3; two patients without AHP, 57-year-old male and 70-year-old female, and one female AHP patient, 84 years of age). The study was approved by the local NHS Ethics Committee.

### 2.2 Assessment of anosognosia and associated disorders

Eight of the 16 patients were classified as having AHP (four women; mean age = 71.63, SD = 16.18 years, age range: 41–88) and eight were classified as right-hemisphere controls (HP group; five women; mean age = 64.75, SD = 12.14 years, age range: 47–78). This classification was based on the Berti, Ladavas, and Della Corte (1996) interview, which includes general questions (e.g., ‘why are you in the hospital?’), followed by specific questions regarding motor ability (e.g., ‘Can you move your left arm?’), and ‘confrontation’ questions (e.g., ‘Please touch my hand with your left hand. Have you done it?’). The interview is scored on a 3-point scale (2 = denial of motor impairment and failure to reach the examiners hand; 1 = denial of motor impairment, but admits to failure to reach examiner hand; and 0 = full acknowledgement of motor deficits), with patients scoring 1 or 2 categorised as anosognosic. The Feinberg, Roane, and Ali (2000) scale was used as a secondary measure of unawareness. The scale consists of 10 items including general self-report items (e.g. ‘Do you have any weakness anywhere?’) and task-related items (e.g., ‘Please try and move your left arm for me. Did you move it?’). Responses were scored by the examiner for each item (0 = completely aware, .5 = partially unaware, and 1 = complete unawareness), and summed to produce an overall ‘Feinberg awareness score’ (0 = complete awareness, 10 = complete unawareness). Finally, body ownership disturbances such as asomatognosia (the inability to recognise one's own body; Cutting, 1978) and somatoparaphrenia (body ownership delusions; Gerstman, 1942) were assessed using the Cutting (1978) questionnaire. Two AHP patients exhibited disturbances of body ownership: one patient manifested somatoparaphrenia (believing that her left arm belonged to her friend), and the other asomatognosia. No other somatic delusions were noted in either group.

### 2.3 Neurological and neuropsychological assessment

Motor strength of the upper and lower limbs was assessed using the Medical Research Council scale (MRC; Guarantors of Brain, 1986). Premorbid intelligence was assessed using the Wechsler Test of Adult Reading (Wechsler, 2001). Orientation in time, space and person, as well as general cognitive functioning, was assessed using the Mini Mental State Examination (MMSE; Folstein, Folstein, & McHugh, 1975). Working memory was assessed using the digit span task from the Wechsler Adult Intelligence Scale III (Wechsler, 1998). Long-term verbal recall was assessed using the 5-item test from the Montreal Cognitive Assessment (MOCA; Nasreddine et al., 2005). Proprioception was assessed with eyes closed by applying small, vertical, controlled movements to three joints (middle finger, wrist and elbow), at three time intervals (correct responses were rated as 0 and incorrect ones as 1) (Vocat et al., 2010). The customary ‘confrontation’ technique was administered to test visual fields and tactile extinction (Bisiach, Vallar, Perani, Papagno, & Berti, 1986). Five subtests of the Behavioural Inattention Test (BIT; Wilson, Cockborn & Halligan, 1987; line crossing, star cancellation, copy, representational drawing and line bisection) were employed to assess unilateral, visuospatial neglect. Personal neglect was assessed using the ‘one item test’ (Bisiach et al., 1986), and the ‘comb/razor’ test (McIntoch, Brodie, Beschin, & Robertson, 2000). Executive and reasoning abilities were assessed using the Frontal Assessment Battery (FAB; Dubois, Slachevsky, Litvan, & Pillon, 2000), and the Cognitive Estimates test (Shallice & Evans, 1978). The Hospital Depression and Anxiety Scale (HADS; Zigmond & Snaith, 1983), was used to assess depression and anxiety.

### 2.4 Experimental study design

Our main experimental aim was to induce positive and negative emotions in patients with AHP and HP controls, and assess their effects on motor awareness. To this end, we administered a standardised cognitive task, the Hayling Sentence Completion Test of executive functioning (Burgess & Shallice, 1997), which entails two similar tasks varying in difficulty. Namely, a simple, sentence completion task (measuring processing speed), and a more difficult sentence completion task, in which patients have to provide responses that are unrelated to the meaning of the sentences (measuring inhibition of automatic responses). Healthy controls and particularly neurological populations are known to perform faster on the first task, and with fewer errors, compared with the second task (Burgess & Shallice, 1997; see Results section below for confirmation of this result in our sample). In order to ensure the induction of positive and negative feelings respectively, we further manipulated the explicit, verbal feedback provided by the experimenter after each trial: positive feedback was provided following trials of the easy task, and negative feedback was provided following trials of the difficult task. Hence, feedback could be administered ‘realistically’ and ensure construct validity. This feedback manipulation can be understood as a mood induction procedure (Nummenmaa & Niemi, 2004), widely used in psychological research, including with neurological patients (e.g., Mograbi, Brown, Salas, & Morris, 2012). The induced emotions are considered short lived and within the normal daily range of emotional experience for most people (Frost & Green, 1982; Isen & Gorgoglione, 1983; Martin, 1990). This was confirmed in this sample at debriefing (see procedures section below).

The experiment had a 2 (Group: AHP *vs* HP) × 2 (Emotion: positive *vs* negative feedback) mixed factorial design, with Emotion as the within-subjects factor. Due to the nature and the standardised administration order of the Hayling Test (Part 1: the easier sentence completion task is followed by Part 2: the harder sentence completion task) positive feedback preceded negative feedback in our experiment. Thus, to examine possible order effects, we also conducted a control experiment in two additionally recruited AHP patients, in whom we reversed the order of positive and negative feedback (i.e., first administering Section 2 with negative feedback, and then Section 1 with positive feedback).

Finally, in order to determine the specificity of the emotion induction on motor awareness we conducted an additional control experiment with three right-hemisphere damaged patients. The experimental procedure was identical to the above, with the exception of additional pre-and-post measures to assess any changes in visuospatial neglect, personal neglect, and anosognosia for drawing neglect, in addition to motor awareness. Specifically, changes in neglect were assessed by administering the copy, line bisection and star cancellation subtests of the BIT (Wilson et al., 1987) and the ‘one-item test’ (Bisiach et al., 1986) pre-and-post the positive and negative emotion induction. Four additional questions were added to the motor awareness questionnaire (please see below) to assess awareness of drawing neglect (Berti et al., 1996). Referring to their performance on the ‘copy’ subtest of the BIT (administered before the experiment; Wilson et al., 1987) patients were asked: (i) two general questions (e.g., “Are you happy with your drawing of the Daisy?” and “Are the daisies alike?”); and (ii) to provide subjective ratings of their drawing performance using a 11-point Likert-type scale (e.g., “Using this scale from 0 to 10, how good is the drawing, 0 being not good at all and 10 being very good?” and “Using this scale from 0 to 10, how alike are the drawings, 0 being not at all alike and 10 being exactly the same?”).

### 2.5 Measures

The primary dependent variable was ‘awareness change’, which was based on a motor awareness questionnaire, developed based on pre-existing, validated measures (e.g., Berti et al., 1996; Marcel et al., 2004), and administered immediately before and after each Emotion condition. Previous studies have suggested that AHP patients may ‘learn’ the ‘correct’ responses to answers on awareness measures when repeatedly administered (Marcel et al., 2004). To avoid such repetition confounds, four equivalent versions of the questionnaire were developed. Each version comprised seven items, covering four domains: (i) two general awareness questions (e.g., “Do you have any weakness anywhere?”); (ii) one question related to left unimanual ability, followed by a ‘confrontation’ and ‘check’ question (e.g., “Can you wave to me with your left hand? Please do it for me now. Have you done it?”); (iii) one question concerning bimanual action ability, each followed by confrontation and check questions (e.g., “Can you tie a knot? Please do it for me now. Have you done it?”), and (iv) one bipedal awareness question (e.g., “Can you climb a ladder?”). Each question was scored according to the method of Feinberg et al. (2000): 0 = awareness; .5 = partial awareness; and 1.0 = unawareness; therefore, higher scores indicated greater unawareness (range = 0–7). For each Emotion condition (i.e., positive and negative feedback), we subtracted the post-induction awareness score from the pre-induction awareness score of each patient, to obtain a main measure of awareness change.

Additionally, in order to evaluate the effects of emotional feedback on patients' emotional state *per se*, patients were asked to provide a subjective rating of their current emotional state on a 6-point Likert-type scale (i.e., “Using this scale from zero to five, zero being very unhappy and five being very happy, how do you feel right now?”). The scale was read aloud to patients and also presented visually as a vertical scale on an A4 sheet of paper (0 at the bottom and 5 at the top), positioned in the patient's right visual field in order to minimise possible unilateral visual neglect effects. Patients were familiarised with the rating scale before the experiment.

### 2.6 Procedures

The experiment was organised into two phases: [i] administration of Hayling Test Part 1 (simple sentence completion) with positive feedback, and [ii] administration of Hayling Test Part 2 (inhibition of automatic response) with negative feedback. These were conducted in a single session, separated by a 30-min interval, during which standard neuropsychological tests (see above) were administered without feedback. Part 1 of the Hayling Test requires the patient to complete a series of sentences with the last word missing from it as fast as possible (e.g., “The rich child attended a private …”, response: school). The response and reaction time are recorded and the total time score is converted into a scaled score. In part 2, the patient is again asked to complete a series of sentences as above, but their response is to be completely unconnected to the sentence (e.g., “London is a very busy …”, possible response: banana). The response and the reaction time are recorded, and the total time and response errors are converted into a scaled score.

Positive feedback was provided in a standardised manner, using one of the following seven statements, in a pseudorandomised order: (i) “Well done”, (ii) “That is correct”, (iii) “Your answer was very quick”, (iv) “Excellent work”, (v) “You are doing so well on this task”, (vi) “Very impressive”, and (vii) “Your performance has been excellent so far”. Positive feedback was matched to performance as much as possible, i.e., most answers were correct and given within one minute and hence one of the above statements was provided. In the unlikely event that an answer was wrong, statement (iii) was provided; or, if an answer was very slow (more than one minute), this statement was not used and one of the other statements were provided. We wish to highlight that, although this feedback was realistic in all cases, it was pre-selected and false in the sense that it did not correspond to the norms of the Hayling Test.

Similarly, negative feedback was provided using one of the following seven standard statements: (i) “That is incorrect”, (ii) “You are not doing very well on this task”, (iii) “Your performance has been very poor so far”, (iv) “That is the wrong answer”, (v) “You are doing poorly so far”, (vi) “Your answer was too slow”, and (vi) “You are not performing very well”. Feedback was consistent with patients' actual performance as much as possible (in the same manner as above, but matched to the poor performance of patients).

Measures of awareness were taken immediately before (i.e., pre-induction awareness) and after (i.e., post-induction awareness) the two parts of the task. The emotion rating scale was completed after each post-induction awareness questionnaire, in order not to influence the latter. During the control experiment, the procedures were identical to the above, except for reversing the order of phases one and two.

Patients were carefully and fully debriefed following completion of the experiment; the purpose of the positive and negative feedback were fully explained, and any questions were addressed. It was stressed that the feedback provided did not reflect of their actual performance on the Hayling Task, as determined by the available, standardised norms, or by the face value impressions the task itself might generate. Any ongoing emotional distress (if experienced) was fully discussed and reflected upon to ensure that the patients' emotional state was stable. There were no particularly strong reactions during the experiment, or following debriefing, and none of the patients reported having guessed or suspected the manipulation.

### 2.7 Statistical analysis

All behavioural analyses were conducted in Stata 11 (StataCorp, 2011). Independent samples *t*-tests were used to analyse mean differences between groups on neuropsychological tests. Items that were not normally distributed were also analysed using the non-parametric equivalent (Mann–Whitney *U* test) to confirm our findings (see Supplementary Materials).

#### 2.7.1 Analysis of main experiment

The differential ‘awareness change’ scores (see Measures) were used as the outcome measure in all analyses, which were conducted using multiple linear regression. The awareness change data were not normally distributed, hence we applied bootstrapping with 1000 repetitions (bootstrapping makes no assumption as to the distribution of the data; Guan, 2003); bootstrapped standard errors (SE) are therefore reported. The same analysis was also run while co-varying for overall negative mood (HADS depression scores, as these were found to differ between the groups, see below). Preliminary examination of the awareness change data identified one HP control patient scoring more than two SD above the group mean, and hence this patient was removed from subsequent experimental analyses as an outlier.

#### 2.7.2 Analysis of control variables

A multiple linear regression (as above) on emotion ratings was used to investigate whether patients experienced a change in their emotional state in the two feedback conditions. The same analysis was also run while co-varying for overall negative mood (HADS depression scores). Furthermore, to ensure there was no difference in the baseline awareness scores preceding the positive and negative feedback conditions (particularly given the fixed order of the task), we conducted non-parametric tests comparing the baseline awareness scores preceding the positive and the negative feedback conditions in each group. In addition, we also compared between groups the total scaled scores of the Hayling Sentence Completion test, as well as the scaled scores for Part 1 and 2, to ensure the actual performance of both groups was consistent with the task's expected difficulty levels, and that the provided feedback was realistic and of similar relevance to both groups. Additionally, modified *t*-tests (SINGLIMS_ES; Crawford, 2010; Crawford et al., 2002, 1998) were used to determine whether the awareness change scores of the two AHP patients in the reverse-order experiment (see Section 2.4) differed significantly from those of the HP group. Finally, in order to investigate whether any changes in awareness resulting from the experiment had a lasting effect, non-parametric tests were used to compare Feinberg awareness scores acquired on initial assessment (prior to the experimental session) with those obtained 1–3 days after the experiment was conducted.

### 2.8 Lesion analysis methods

Routinely acquired clinical CT (*n* = 10) and MRI (*n* = 5) data sets were obtained within the first week of admission [admission to neuroimaging interval: mean = 4.26 days, SD = 4.88 days]. The clinical data set of one HP control patient was unavailable and the patient was therefore excluded from further imaging analyses. Available structural data were converted into software-readable formats for further processing. To facilitate comparison between the clinical data and a standard space template, we manually reoriented the native structural scan of each patient to the origin of the template using SPM (Statistical Parametric Mapping, http://www.fil.ion.ucl.ac.uk/spm/). Lesions were then reconstructed onto the MNI (Montreal Neurological Institute) template provided within MRIcron (http://www.mccauslandcenter.sc.edu/mricro/mricron/) whilst using all available clinical scans to guide the delineation. Lesions were mapped by two researchers, who were blind to group classification and the behavioural scores of the patients.

In a first step, lesion volume was obtained. Subsequently, percentage lesion overlay maps for both groups, AHP and HP, were computed in FSL (FMRIB Software Library, http://fsl.fmrib.ox.ac.uk/fsl/fslwiki/). In a second step, a lesion difference map between both groups was computed.

The classical voxel-based lesion-symptom mapping (VLSM) approach (Bates et al., 2003; Rorden et al., 2007) as implemented in the software package NPM (non-parametric mapping; http://www.cabiatl.com/mricro/npm/) (Karnath, Berger, Küker, & Rorden, 2004; Rorden & Karnath, 2004) was used to identify anatomical regions associated with: i) the presence of anosognosia (Feinberg awareness scores, inverted to adhere with the NPM prerequisite of the directionality of the input data) and ii) the awareness change induced by the experimental design (‘change in awareness' scores). Results were calculated with the permutated non-parametric Brunner–Menzel test to correct for multiple comparison and small sample size (Rorden et al., 2007; Volle et al., 2011). Results were then projected onto a high-resolution template (Holmes et al., 1998) in MNI standard space using MRIcron.

## 3 Results

### 3.1 Demographic and neuropsychological results

Patients' demographic characteristics and their performance on standardised neuropsychological tests are summarised in Table 1. The groups did not differ significantly in terms of age, education or symptom onset to assessment interval. As expected, there was a significant difference in awareness scores between the AHP and HP groups on both the Berti et al. (1996) interview [*t*(14) = 5.60, *p* = .00] and the Feinberg et al. (2000) scale [*t*(14) = 7.06, *p* = .00]. The groups showed similar sensory deficits, as well as similar impairments in general cognitive functioning, abstract thinking, reasoning abilities and neglect. Although both groups showed deficits in proprioception, the AHP group was significantly more impaired [*t*(12) = 2.33, *p* = .04]. The AHP group showed significantly lower scores for depression on the HADS when compared to controls [*t*(14) = 3.06, *p* = .01]. This difference was taken into account in subsequent analyses.

### 3.2 Main experimental results: awareness change

**Fig. 1:**
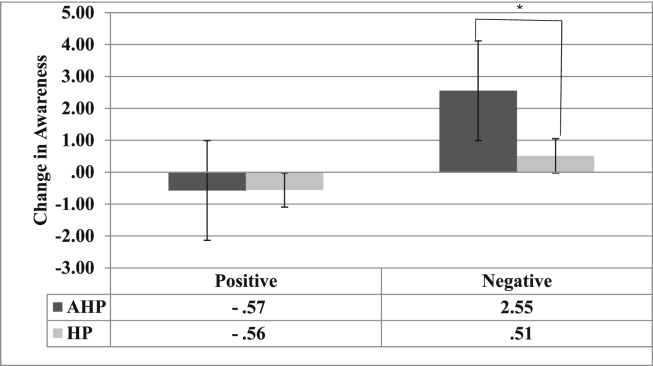
Marginal means and interquartile range (error bars) of the change in awareness for the AHP (dark grey bars) and HP (light grey bars) groups after the positive and negative emotional induction: *p < .05. The *Y*-axis indicates the change in awareness scores analysed by calculating the difference in awareness scores between each condition (post minus pre) for each group. Positive scores indicate an increase in awareness (i.e., less anosognosia) and negative scores indicate a decrease in awareness (i.e., more anosognosia).

A linear regression analysis revealed a significant main effect for the factor Group (*b* = 2.04, SE = −.45, *p* < .001, 95% CI = 1.16; 2.92), with the AHP group showing a greater change in awareness (marginal mean = .99) compared with the HP group (marginal mean = −.02). Also, a significant main effect of Emotion induction type (*b* = −1.07, SE = .46, *p* = .019, CI = −1.96; −.18) was observed, with awareness change being significantly greater following the negative (marginal mean = 1.6) compared with the positive emotional induction (marginal mean = −.57). The interaction between Emotion induction type and Group was also significant (*b* = −2.05, SE = .61, *p* = .001, CI: −3.26; −.84; see Fig. 1), with the AHP group (marginal mean = 2.55) showing a greater change in awareness compared with the HP group (marginal mean = .75) following the negative emotional induction only. Taking the HADS depression scores into account in this analysis did not change the pattern of these results.

A qualitative example of the change in motor awareness observed as a result of the emotion induction is described here. During the pre-awareness assessment one patient stated “No, I have no weakness anywhere, no”, claiming that “I can move my arm, no problem” and was adamant that she raised her left arm and clapped her hands. Following the negative emotion induction, the same patient admitted that her left arm “is not as strong as before the stroke”, saying “I don't think I can move this arm now, it feels weak”. When asked if she can tie a knot, she replied “I'm not so sure now” and after attempting the action, she observed “no, I can't do that.”

### 3.3 Emotional state induction

**Fig. 2:**
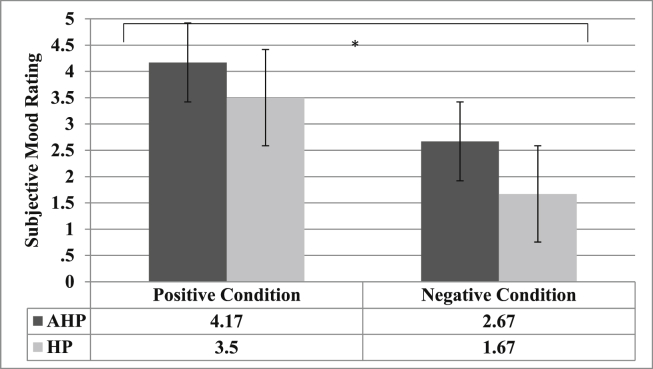
Marginal means and interquartile range (error bars) of emotion ratings for AHP (Dark grey bars) and HP (light grey bars) groups after positive and negative mood induction: *p < .05. The *Y*-axis indicates the patient's subjective mood ratings on a scale from zero to five (0 = very unhappy; 5 = very happy).

To investigate whether patients experienced a change in their emotional state following the positive and negative induction respectively, we examined the main effects of Emotion (positive *vs* negative feedback) and Group (AHP *vs* HP) on emotion ratings. The regression analysis confirmed a main effect of Emotion (*b* = 1.83, SE = .439, *p* < .001, CI: .97; 2.69) with patients giving significantly lower emotion ratings (i.e., reporting feeling less happy) following the negative emotional induction (marginal mean = 2.17) compared with the positive emotional induction (marginal mean = 3.83). The model also showed that the factor Group significantly predicted emotion ratings (*b* = .99, SE = .49, *p* = .046, CI: .019; 1.97), with AHP patients showing more positive emotion ratings (marginal mean = 3.41) compared with right-hemisphere controls (marginal means = 2.59). However, there was no significant interaction between the factors induction type and group (*b* = −.33, SE = .64, *p* = .6, CI: −1.59; .93; see Fig. 2).

### 3.4 Baseline awareness scores

A Wilcoxon Signed Rank Test revealed that there was no significant difference between pre-awareness scores of the positive (median = 2) and of the negative condition overall (median = 3, *Z* = −.27, *p* = .82, *r* = .067). This applied also to the AHP group (*Z* = −.9, *p* = .563, *r* = .23) and the HP group (*Z* = −.7, *p* = .75, *r* = .18), in respective, separate analyses.

### 3.5 Performance on the Hayling Test

Analysis of the Hayling Sentence Completion Test using a Mann–Whitney *U* test showed no significant difference between total scaled scores of the AHP and HP groups (*Z* = −1.14, *p* = .28, *r* = .29). According to the tests norms, overall scaled scores indicated that the AHP group's performance was ‘low average’ (median = 4), while the HP group's performance was ‘moderate average’ (median = 5). Similarly, there was no difference found in Hayling part 1 (*Z* = −.9, *p* = .42, *r* = .23), with the scaled score for completion time being ‘low average’ for the AHP group (median = 4) and ‘moderate average’ for the HP group (median = 5). This again applied to Hayling part 2, with no difference found between groups in their total scaled score for completion time (*Z* = −.4, *p* = .8, *r* = .1) and response errors (*Z* = −1.1, *p* = .31, *r* = .28), with the AHP group performing ‘average’ for time (median = 6) and ‘abnormal’ for response errors (median = 1.5). Similarly, the HP group performed ‘average’ for time (median = 6) and ‘abnormal’ for responses errors (median = 2) (see Supplementary Materials). Therefore, the feedback given was realistic based on patients' actual performance, with both groups performing better on part 1 than on part 2, and showing no differences between groups on either part.

### 3.6 Reverse order control condition

The two AHP patients who performed the experiment in the reverse order showed the same pattern of results as found in the main group analysis. After the negative emotion induction, both patients showed a greater improvement in awareness (AHP09: mean = 5, AHP10: mean = 3.5) compared to the control group (mean = .5; SD = .82; AHP09: *t*(7) = 5.13, *p* = .001, *r* = 5.49; AHP10: *t*(7) = 3,42, *p* = .007, *r* = 3.66). There was no difference between either AHP patient and the HP control group in awareness change following positive emotion induction (AHP09: *t*(7) = .45, *p* = .33, *r* = .48; and AHP10: *t*(7) = 1.7, *p* = .07, *r* = 1.81).

### 3.7 Specificity of effect control condition

The three patients with right-hemisphere damage who performed this additional control experiment showed no change in personal neglect assessments, and a minor change in visuospatial neglect, with extrapersonal neglect becoming slightly worse following negative versus positive induction in two patients. Additionally, there was a non-mood specific improvement in awareness of neglect in one patient. The results are summarised in 3 case reports below (see Supplementary Materials Table S2 for a summary of results).

Patient HP09 presented with no AHP, no personal neglect, no visuospatial neglect except on the ‘copy’ subtest, and mild unawareness of drawing neglect. There was no change in visuospatial and personal neglect, or awareness of drawing neglect following the positive and negative emotion induction condition. Patient HP10 presented with no AHP, mild personal neglect, visuospatial neglect and unawareness of drawing neglect. She showed no change in the line bisection subtest, personal neglect scores, and general questions for awareness of drawing neglect, but a small increase in visuospatial neglect following the positive and negative emotion induction conditions. There was also a small increase in awareness of drawing neglect following the negative emotion induction, but a much larger increase in awareness following positive induction. Lastly, patient AHP11 presented with AHP, personal neglect, visuospatial neglect and mild unawareness of drawing neglect. There was no change in her personal neglect and awareness of drawing neglect scores, and no change in her performance on the line bisection subtest following the negative and positive emotion inductions. There was a small increase in visuospatial neglect (star cancellation subtest) following the negative but not positive emotion induction.”

### 3.8 Follow-up awareness testing

Wilcoxon signed rank tests showed that there was no significant difference in Feinberg awareness scores before and after the experiment, in either the AHP (*Z* = −.45, *p* = .66, *r* = .12) or HP group (*Z* = −1.63, *p* = .1, *r* = .42), suggesting that the observed awareness changes were temporary and experimental effects, rather than permanent, clinical changes.

### 3.9 Lesion analysis

**Fig. 3:**
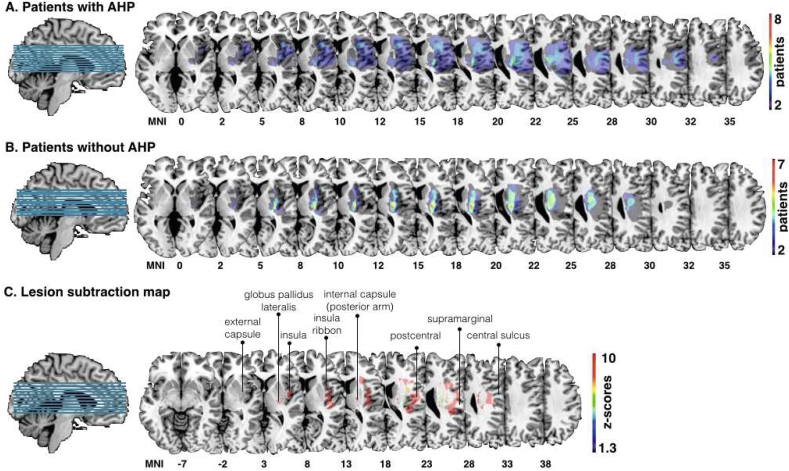
Group-level lesion overlay maps for patients with anosognosia for hemiplegia (AHP) and controls. A. Overlay of lesions in patients with anosognosia (AHP; *n* = 8); B. Overlay of patients without anosognosia (*n* = 7). C. Statistical analysis comparing the two populations of patients (AHP present-AHP absent; results are corrected for multiple comparisons, *p* < .05 for *Z* > 1.3).

All lesions resulted from a first-ever unilateral stroke, mainly within the right middle cerebral artery territory. Group-level percentage lesion overlay for the AHP group (*n* = 8) identified the involvement of cortical and subcortical areas, comprising the inferior and superior frontal gyri, the pericentral cortex, the insula and insula ribbon, and the internal capsule (see Fig. 3A). In comparison, the lesion overlap map for the HP group (*n* = 7) revealed a more focal lesion pattern involving mainly subcortical regions (see Fig. 3B). Lesion volume (defined by number of voxels) was not significantly different between the AHP group (mean = 37132.5, SD = 43782.65) and the HP group (mean = 25997.14, SD = 33536.03; *t* (15) = .55, *p* = .594). The lesion subtraction map identified mainly the anterior and posterior insular ribbon, the posterior basal ganglia, and dorsal pericentral areas to differ between the groups (see Fig. 3C).

**Fig. 4:**
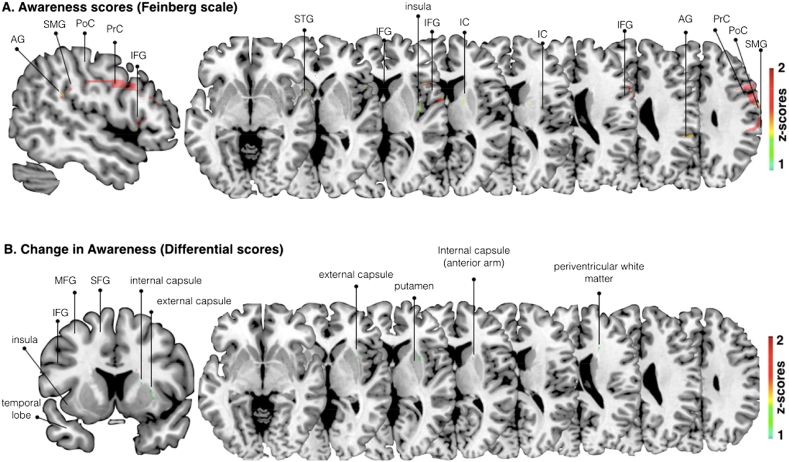
Voxel-based (topological) lesion-deficit analysis. A. Damaged MNI voxels predicting the severity of unawareness of symptom deficits when co-varying for lesion size (Feinberg scale, inverted, continuous measure; *p* < .05 for *Z* > 1.6449). B. Damaged MNI voxels predicting the change in awareness (differential scores, pre and post mood induction) when co-varying for lesion size (continuous measure; *p* < .05 for *Z* > 1.6449). PrC = precentral, PoC = postcentral, SMG = supramarginal, STG + superior temporal gyrus, IFG = inferior frontal gyrus, IC = internal capsule, MFG, middle frontal gyrus.

VLSM analysis using the continuous Feinberg awareness scores, revealed that voxels within the posterior insula, the supramarginal, the angular and superior temporal gyrus (SMG, AG and STG), internal capsule, pericentral gyri, and the inferior frontal gyrus (IFG) were significantly associated with differences in awareness (*p* < .05) (see Fig. 4A). Similar results were found when co-varying lesion size. Additionally, VLSM analysis, looking at the experimental change in awareness scores (i.e., differential scores following negative emotional induction only), without and with co-variation of lesion size, identified significant voxels (*p* < .05) within the anterior arm of the internal capsule, the anterior insula, the anterior lateral putamen with a lateral extension into the external capsule and an additional region in the dorsal anterior periventricular white matter (likely to contain limbic white matter connections) (see Fig. 4B).

## 4 Discussion

In the present study, we experimentally induced positive and negative emotions in patients with AHP and HP controls, and measured the resulting changes in motor awareness. We also investigated the brain lesions associated with the clinical diagnosis of AHP, as well as with performance on our experimental task. The main behavioural finding was that patients with AHP showed a significant improvement in motor awareness following a negative, but not a positive, emotion induction. The main finding of the analysis combining experimental and lesion data was that lesions to the putamen, the anterior insula, the capsules and the anterior periventricular white matter were associated with less awareness improvement on our experimental task. These findings are discussed in turn below.

To our knowledge, this is the first experimental demonstration of the role of emotion in AHP. Our results show that negative, self-referential emotion induced by social feedback can lead to temporary improvements in motor awareness, in patients who otherwise show stable AHP. These results are consistent with previous clinical observations of transitory awareness improvements and ‘catastrophic reactions’ following discussions of negative themes such as loss, separation or mortality (Kaplan-Solms & Solms, 2000). They are also consistent with experimental manipulations of perspective-taking, in which taking a third person perspective of one's disability can lead to awareness improvements and increase of depressive emotions (Fotopoulou et al., 2009; Marcel et al., 2004). We believe these results cannot be accounted for by either the psychodynamic or ‘valence’ hypothesis (see Introduction), and instead are best explained by theories that assume ‘top-down’, emotional abnormalities (Fotopoulou et al., 2010, 2014; Vuillemier, 2004; Turnbull et al., 2005). Moreover, although we tested changes to neglect and unawareness for neglect following emotion induction in only a small subset of patients, it appears that the effects of negative emotion on awareness are specific to motor awareness and do not extend to neglect or its unawareness. We discuss these findings and their potential interpretations in turn below.

While our results could be interpreted as psychodynamic ‘lifting’ of denial and repression, the psychodynamic hypothesis could just as easily predict the opposite result, namely a defensive, decrease of awareness due to the negative emotions experienced following negative feedback. Thus, the predictions of this theory in relation to our results are not clear. Similarly, although patients with AHP showed significantly less depressive feelings and symptoms than controls on a self-report measure (see also Fotopoulou et al., 2010), our experimental results could not be accounted for by the ‘valence’ hypothesis. This is because patients with AHP showed greater awareness changes following the negative emotion induction, suggesting that they were able to process such emotions at some level. Indeed, both groups reported feeling more negative emotions following negative versus positive feedback in a ‘manipulation check’ measure. Interestingly, during the experiment, patients with AHP reported feeling overall more positive emotion than control patients, but this effect was unrelated to the valence of the feedback provided. This may relate to the aforementioned, more general tendency of patients with AHP to report (rather than experience) less negative emotions (see also Turnbull et al., 2005). Thus, as our patients were able to experience increased negative emotions following the negative emotion induction and increased positive emotions following the positive emotion induction, our results suggest that their emotional difficulties do not consist of a primary deficit in emotional processing (as the valence hypothesis suggests). Instead, as their emotional difficulties seem to relate more specifically to their motor awareness (see also above), they may be suffering from a more specific, higher-order impairment in consciously, self-attributing negative emotions, i.e., attributing negative emotions to at least some of their higher-order self-representations (see also Fotopoulou, 2010; Turnbull et al., 2005).

This interpretation is also supported by the findings of our lesion mapping analysis. Specifically, the presence (lesion overlay results) and severity (Feinberg VLSM results) of anosognosia were associated with lesions to a range of cortical and subcortical areas previously associated with AHP (Berti et al., 2005; Fotopoulou et al., 2010; Karnath et al., 2005; Moro et al., 2011; Vocat et al., 2010). However, worse performance on the critical condition of our experimental task (i.e., less awareness change following negative feedback) was associated with lesions to the putamen, the anterior insula, the capsules and the anterior periventricular white matter.

The insula, and particularly its anterior sectors, is increasingly identified as the neural substrate for the conscious representational of internal bodily signals (interoception; Critchley, Wiens, Rotshtein, Öhman, & Dolan, 2004; Craig, 2009), as well as for the processing of salience (Seeley et al., 2007). Thus, in patients with AHP, damage to the right insula and related white matter connections may be linked with impoverished interoceptive signals about the left-side of the body (see also Fotopoulou et al., 2010; Karnath et al. 2005). We speculate that this deficit may affect how patients process the salience and emotional significance of signals arising in this body side, thus explaining how they can remain in denial of their paralysis and/or apathetic towards the normally alarming sight of a paralysed left arm (Romano, Gandola, Bottini, & Maravita, 2014). Similarly, the functional role of the basal ganglia and particularly the striatum has been associated with prediction error-driven learning (O'Doherty et al., 2003), as well as the aberrant salience theories of psychosis (Gray et al., 1991; Kapur, 2003). In AHP such deficits can be linked with both specific instances of aberrant motor monitoring in functionally specialised systems (Berti et al., 2005), or more generally in global error monitoring, salience processing and belief updating (Davies, Davies, & Coltheart, 2005; Venneri & Shanks, 2004; Vocat et al., 2013). For example, according to a probabilistic, predictive coding theory of AHP (Fotopoulou, 2012; 2014), such lesions could be understood to disrupt neuromodulatory circuits in AHP, leading for example to dopamine-depletion and a difficulty in optimising the precision (uncertainty) of prediction errors (Friston et al., 2012), affecting their salience and, ultimately, the learning of new information. Thus, even when signals about the current state of the body may be available, they may be ‘imprecise’, and thus unable to update prior beliefs about the self. This ultimately leads to aberrant inferences about one's current abilities and abnormal adherence to past beliefs about the body.

We can thus speculate that in AHP patients who fail to update their emotions and beliefs about their current state of the body (i.e., their left-sided paralysis), the provision of negative feedback by social means can generate negative emotions about the self and new learning on the basis of other intact areas. Future studies will be needed to verify this prediction, perhaps using functional neuroimaging to detect residual emotional processing in AHP patients. In addition, given the potential specificity of our effects (concerning motor but not spatial awareness), future studies should explore the psychological and neural relation between emotional processing and the motor system. Indeed, a growing literature is suggesting a tight interrelation between emotion and motor representations (see Pereira et al., 2010; Gentsch & Synofzik, 2014. Consistent with the current findings, previous studies have shown that while negative emotional processing competes for attentional resources with visual tasks to the detriment of performance on the latter (Erthal et al., 2005; Hartikainen, Ogawa,& Knight, 2000; Tipples & Sharma, 2000), they may enhance processing in motor-related brain areas. Indeed, several studies of non-human primates have found the involvement of motor-related cortical areas during threatening contexts (e.g., Graziano & Cooke, 2006), while emotional threat has been found to be associated with increased motor cortex excitability in humans (Baumgartner, Willi, & Jäncke, 2007; Hajcak et al., 2007; Oliveri et al., 2003). Induction of fear has been found to modulate activity in primary motor cortex and putamen (Butler et al., 2007; Phelps et al., 2001). These findings have been interpreted in contemporary theories of emotion as consistent with the idea that aversive contexts engage motor circuits in order to prepare participants for action that may protect the organism from threat (Azevedo et al., 2005; Bradley, Codispoti, Cuthbert, & Lang, 2001; Hajcak et al., 2007). The current results may indeed relate to such an enhancement of activity in residual motor-related areas and future, electromyography or neuroimaging studies can specifically test such speculations and predictions.

### 4.1 Limitations

Our small sample size and the inherent limitations of the voxel-based lesion-symptom mapping approach (Geva, 2012; Rorden, 2007; Volle et al., 2011), only allow for preliminary evidence of the possible neural correlates observed. Nevertheless, our VLSM approach, compared to other lesion analysis methods, does offer several advantages, including the use of continuous scores of behavioural performance instead of the classification of patients into categorical groups. An additional limitation concerns the fact that we did not include a ‘neutral emotion’ or ‘no feedback’ control condition in our experiment, which we could compare with both negative and positive emotion conditions. In addition, we could not control for floor effects in the control group given the unique nature of anosognosia. Nevertheless, although there was a smaller margin for change in awareness scores for the control group, there was still a small change evident in the same direction as the AHP group. Furthermore, this control group allowed us to control for other more basic confounding effects such as age, test adherence, cognitive functioning, practice, repetition, comprehension and fatigue effects.

Importantly, the observed changes were temporary and generated under specific experimental conditions, and thus the results of our experiment are not directly relevant to clinical studies. However, our findings do have indirect implications for clinical work; they reinforce the previously demonstrated link between awareness improvement and depressive feelings, as well as more generally emphasise the role of emotion in the syndrome, despite some patients' apparent lack of emotional reactivity.

### 4.2 Conclusion

To our knowledge, this is the first study to conduct a systematic, experimental investigation of the relation between emotion and motor awareness in right-hemisphere stroke patients with AHP. We have shown that motor awareness is sensitive to the induction of negative emotions in a social context, and this effect seems to relate to insular and striatal areas, and related white matter connections. We argued that neither pure psychodynamic, nor neurocognitive theories are sufficient to explain these results. Instead, we speculatively suggest that lesions to such regions may impair interceptive signals and neuromodulatory pathways associated with motivation. Ultimately, such deficits result in an inability to update prior beliefs about the self and affectively personalise new sensorimotor information.

## Funding

This work was funded by an European Research Council (ERC) Starting Investigator Award for the project ‘The Bodily Self’ N°313755 to A.F., a Neuropsychoanalysis Foundation Fellowship to PMJ., and a Commonwealth Scholarship, an Oppenheimer Memorial Trust Fellowship, and a Neuropsychology International Fellowship Award from the British Psychological Society in conjunction with the British Neuropsychological Society to S.B.

## Figures and Tables

**Table 1:** Groups' demographic characteristics and neuropsychological profile.

	AHP(*n* = 8)		HP(*n* = 8)		*t*-Test		
	Mean	SD	Mean	SD	*t*	df	*p*
Age (years)	71.63	16.18	64.75	12.14	.96	14.00	.35
Education (years)	11.88	1.81	12.63	1.92	.68	14.00	.51
Days from onset	11.13	11.26	14.38	10.56	.60	14.00	.56
MRC Left upper limb	.25	.46	.38	.52	.51	14.00	.62
MRC left lower limb	.63	.92	1.00	1.07	.75	14.00	.46
Premorbid IQ-WTAR	41.50	7.79	33.00	7.62	1.41	10.00	.19
Berti awareness interview	1.63	.52	.25	.46	5.60	14.00	.00*
Feinberg awareness scale	6.31	2.17	.63	.69	7.06	14.00	.00*
Orientation	2.88	.35	3.00	.00	1.00	7.00	.35
Digit span forwards	5.63	1.19	6.13	.99	.91	14.00	.38
Digit span backwards	2.88	.83	3.38	1.30	.91	14.00	.38
MOCA memory	3.75	.89	4.17	.98	.83	12.00	.42
MMSE	22.20	6.02	25.00	2.16	.88	7.00	.41
Visual fields	4.29^a^	1.89	3.57^a^	1.99	.69	12.00	.50
Somatosensory (max 6)	3.38^a^	1.41	3.00^a^	1.60	.50	14.00	.63
Proprioception (max 9)	3.71	2.21	6.57	2.37	2.33	12.00	.04*
Comb/razor test left	4.75	4.13	5.25	2.60	.29	14.00	.78
Comb/razor test right	12.63	5.10	10.63	2.97	.96	14.00	.35
Comb/razor test ambiguous	5.88	1.96	4.13	2.42	1.59	14.00	.13
Bisiach one item test	.75	.46	.38	.52	1.53	14.00	.15
Line crossing right	11.50	6.44	16.25	2.05	1.99	8.41	.08
Line crossing left	6.75^a^	8.14	10.00^a^	8.68	.77	14.00	.45
Star cancelation right (omissions)	13.75	6.11	11.00	6.19	.89	14.00	.39
Star cancelation left (omissions)	21.25^a^	10.43	18.88^a^	10.86	.45	14.00	.66
Copy	.50^a^	.76	1.00^a^	1.07	1.08	14.00	.30
Representational drawing	.25	.46	.50	.53	1.00	14.00	.33
Line bisection right	.43^a^	.53	.38^a^	.52	.20	13.00	.85
Line bisection centre	.57^a^	.53	.75^a^	.46	.69	13.00	.50
Line bisection left	.38^a^	.52	.50^a^	.53	.48	14.00	.64
Cognitive estimates	16.71^a^	4.86	15.50^a^	2.26	.56	11.00	.59
FAB total score	11.40^a^	2.70	13.50^a^	2.51	1.43	11.00	.18
HADS depression	2.88	2.70	8.00^a^	3.89	3.06	14.00	.01*
HADS anxiety	5.13	3.00	7.25	4.89	1.05	14.00	.31

Berti awareness interview = Berti et al. (1996); Feinberg Awareness scale = Feinberg et al. (2000); MRC = Medical Research Council (Guarantors of Brain, 1986); MOCA = The Montreal Cognitive Assessment (Nasreddine et al., 2005); Comb/razor test = tests of personal neglect (MacIntoch, Brodie, & Beschin, 2000); Bisiach one item test = test of personal neglect; Visual fields and somatosensory = customary ‘confrontation’ technique = Bisiach, Vallar, & Perani (1986); line crossing, star cancellation, copy & representational drawing = conventional sub-tests of Behavioural Inattention Test (Wilson, Cockborn & Halligan, 1987); FAB = Frontal Assessment Battery (Dubois et al., 2000); HADS = Hospital Anxiety and Depression scale (Zigmond & Snaith, 1983). *Significant difference between groups, *p* < .05.

a Scores below tests' cut-off points, or more than 1 SD below average mean.
