# Corrigendum to “The affective modulation of motor awareness in anosognosia for hemiplegia: Behavioural and lesion evidence” [Cortex 61 (2014) 127–140]

**DOI:** 10.1016/j.cortex.2015.01.001

**Published:** 2015-05

**Authors:** Sahba Besharati, Stephanie J. Forkel, Michael Kopelman, Mark Solms, Paul M. Jenkinson, Aikaterini Fotopoulou

**Affiliations:** a King's College London, Institute of Psychiatry, UK; b Department of Psychology, University of Cape Town, South Africa; c Clinical, Educational & Health Psychology, Division of Psychology & Language Sciences, University College London, UK; d King's College London, Department of Neuroimaging, Natbrainlab, Institute of Psychiatry, UK; e Department of Psychology, School of Life and Medical Sciences, University of Hertfordshire, UK

The authors regret that some errors existed in the anatomical labels in Figure 4. The corrected figure can be seen below.

**Figure 4.**
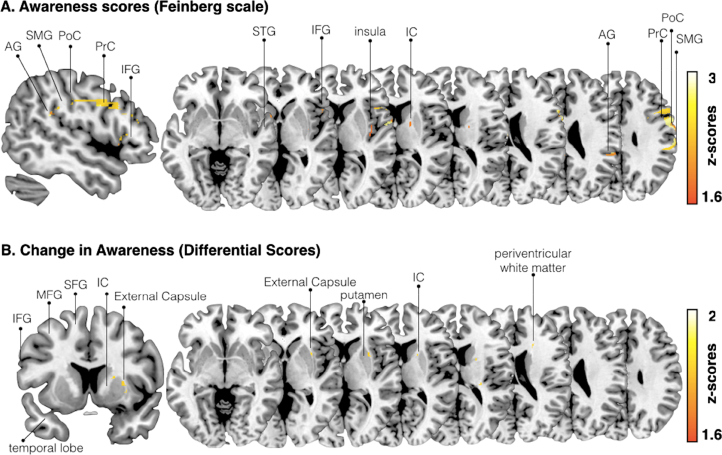
Voxel-based (topological) lesion-deficit analysis. **A**. Damaged MNI voxels predicting the severity of unawareness of symptom deficits when covarying for lesion size (Feinberg scale, inverted, continuous measure; p <0.05 for Z >1.6449). **B**. Damaged MNI voxels predicting the change in awareness (differential scores, pre and post mood induction) when covarying for lesion size (continuous measure; p<0.05 for Z >1.6449). PrC = precentral, PoC = postcentral, SMG = supramarginal, STG = superior temporal gyrus, IFG = inferior frontal gryus, IC = internal capsule, MFG, middle frontal gyrus.

The authors would like to apologise for any inconvenience caused.

