# Supplementary material for: The affective modulation of motor awareness in anosognosia for hemiplegia: Behavioural and lesion evidence
Source: Cortex. 2014 Dec;61:127–40. doi: 10.1016/j.cortex.2014.08.016 (PMC4296216; doi:10.1016/j.cortex.2014.08.016)
Supplement: Supplementary file 1 [file mmc1.docx]

**Supplementary Materials**

**Table 1. Groups’ demographic characteristics and neuropsychological profile using non-parametric analysis.**

|  | **AHP(n=8)** | | **HP(n=8)** | | **Mann-Whitney Test** | |
| --- | --- | --- | --- | --- | --- | --- |
|  | **Median** | **Range** | **Median** | **Range** | **Z-Score** | ***P*** |
| Berti awareness left | 2 | 1 | 0 | 1 | -3.23 | 0.00 |
| MRC left upper limb | 0 | 1 | 0 | 1 | -0.52 | 1.00 |
| MRC left lower limb | 0 | 2 | 1 | 3 | -0.79 | 0.49 |
| Digit Span Forwards | 6 | 3 | 6 | 3 | -0.89 | 0.52 |
| MOCA memory | 3.5 | 2 | 4.5 | 2 | -0.83 | 0.56 |
| Comb/razor test left | 3.5 | 14 | 4.5 | 7 | -0.69 | 0.53 |
| Comb/razor test ambiguous | 5.5 | 5 | 4 | 8 | -1.41 | 0.18 |
| Bisiach one item test | 1 | 1 | 0 | 1 | -1.46 | 0.31 |
| Copy | 0 | 2 | 1 | 3 | -1.03 | 0.39 |
| Cognitive estimates | 18 | 14 | 16 | 6 | -0.94 | 0.37 |
|  |  |  |  |  |  |  |

Berti awareness interview= Berti et al. (1996); MRC= Medical Research Council (Guarantors of Brain, 1986); MOCA=The Montreal Cognitive Assessment (Nasreddine, 2005); Comb/razor test = tests of personal neglect (MacIntoch, Brodie, & Beschin, 2000); Bisiach one item test= test of personal neglect; Copy = conventional sub-test of Behavioural Inattention Test (Wilson, Cockborn & Halligan, 1987).

^a^ Scores below tests’ cut-off points, or more than 1 SD below average mean.

*Significant difference between groups, *P*<0.05

| **Modality & Test** | **Patient** | **Positive** | | **Negative** | |
| --- | --- | --- | --- | --- | --- |
|  |  | **Pre** | **Post** | **Pre** | **Post** |
| **Visuospatial neglect** |  |  |  |  |  |
| Line Bisection  (Range:0-3) |  |  |  |  |  |
|  | HP09 | 3 | 3 | 3 | 3 |
|  | HP10 | 0 | 0 | 0 | 0 |
|  | AHP11 | 0 | 0 | 0 | 0 |
| **Visuospatial neglect** |  |  |  |  |  |
| Star cancelation  (54 omissions) |  |  |  |  |  |
|  | HP09 | 2 | 2 | 2 | 2 |
|  | HP10 | 30 | 28 | 26 | 28 |
|  | AHP11 | 50 | 50 | 49 | 50 |
| **Personal neglect** |  |  |  |  |  |
| One item Test  (Range:0-3) |  |  |  |  |  |
|  | HP09 | 3 | 3 | 3 | 3 |
|  | HP10 | 2 | 2 | 2 | 2 |
|  | AHP11 | 1 | 1 | 1 | 1 |
| **Awareness of drawing neglect** |  |  |  |  |  |
| General questions  (Range:0-2) |  |  |  |  |  |
|  | HP09 | 0.5 | 0.5 | 0.5 | 0.5 |
|  | HP10 | 0.5 | 0.5 | 0.5 | 0.5 |
|  | AHP11 | 0 | 0 | 0 | 0 |
| **Awareness of drawing neglect** |  |  |  |  |  |
| Rating scale  (Range:0-20) |  |  |  |  |  |
|  | HP09 | 4 | 4 | 5 | 5 |
|  | HP10 | 14 | 3 | 15 | 12 |
|  | AHP11 | 5 | 5 | 6 | 6 |

**Table 2.** Experimental results of additional control experiment with 3 right-hemisphere brain damaged patients investigating change in visuospatial neglect, personal neglect and awareness of drawing neglect.

_Bisiach one item test= test of personal neglect (Bisiach, Vallar, & Perani (1986); line bisection, star cancellation & copy & = conventional sub-tests of Behavioural Inattention Test (Wilson, Cockborn & Halligan, 1987)._
